# Women and birth partners’ experiences of cervical ripening at home and in hospital

**DOI:** 10.1186/s12884-024-06936-8

**Published:** 2025-01-30

**Authors:** Cassandra Yuill, Mairi Harkness, Helen Cheyne, Boo Charkin, Monica Ferreira, Eloise Price, Amarnath Bhide, Mairead Black, Kathleen Boyd, Neelam Heera-Shergill, Neena Modi, John Norrie, Dharmintra Pasupathy, Julia Sanders, Sarah J. Stock, Rosemary Townsend, Linda J. Williams, Christine McCourt

**Affiliations:** 1 Centre for Maternal and Child Health Research, City St George’s, University of London, Myddelton Street Building, 1 Myddelton Street, London, EC1R 1UB United Kingdom; 2 Nursing Midwifery and Allied Health Professionals Research Unit (NMHAP-RU), University of Stirling, Stirling, United Kingdom; 3 Department in Obstetrics and Fetal Medicine, St George’s Hospital, London, United Kingdom; 4 School of Medicine, Medical Sciences and Nutrition, University of Aberdeen, Aberdeen, United Kingdom; 5 School of Health and Wellbeing, University of Glasgow, Glasgow, United Kingdom; 6 Cysters (CIO), Birmingham, United Kingdom; 7 Faculty of Medicine, Imperial College London, London, United Kingdom; 8 Edinburgh Clinical Trials Unit, Usher Institute, University of Edinburgh, Edinburgh, United Kingdom; 9 Reproduction and Perinatal Centre, Faculty of Medicine and Health, University of Sydney, Sydney, Australia; 10 School of Healthcare Sciences, University of Cardiff, Cardiff, United Kingdom; 11 Centre for Reproductive Health, Institute for Regeneration and Repair, University of Edinburgh, Edinburgh, United Kingdom

**Keywords:** Home cervical ripening, Induction of labour, Outpatient induction of labour, Informed choice, Experience, Acceptability

## Abstract

**Background:**

In the United Kingdom, induction of labour rates are rapidly rising, and around a third of pregnant women undergo the procedure. The first stage, cervical ripening, traditionally carried out in hospital, is increasingly offered outpatient – or ‘at home’. The current induction of labour rates place considerable demand on maternity services and impact women’s experiences of care, and at home cervical ripening has been suggested as potential solution for alleviating these. However, there is a lack of evidence on both women’s and birth partners’ experiences and acceptability of at home cervical ripening informing its practice.

**Methods:**

We undertook a qualitative study of women and their birth partners’ experiences of cervical ripening at home and in hospital. Semi-structured interviews explored experiences, acceptability and consequences of cervical ripening.

**Results:**

We identified six key themes: ‘Information and choice’; ‘Physical and sensorial environments’; ‘Pain’; ‘Uncertainty’; ‘Care during induction’; ‘Lasting effects’. Women and birth partners experienced limited choice about cervical ripening. Many reported that shared hospital spaces contributed to negative experiences, while home environments were comforting. Women were unprepared for cervical ripening-associated pain, and delays and uncertainty during induction caused anxiety. Supportive care contributed to more positive experiences; however, some reported difficult or traumatic experiences related to induction.

**Conclusions:**

Most participants were positive about home cervical ripening, yet our study highlights the lack of information and genuine choice regarding cervical ripening and induction. Privacy, presence of birth partners and supportive care contributed to more positive experiences among women. Home cervical ripening may be acceptable to some women and birth partners in the context of informed choice and personalised care.

## Introduction

For an increasing number of pregnant women who give birth, labour will be started artificially through a process known as induction of labour (IOL). It is offered when the risks to maternal and/or fetal morbidity and mortality are considered greater if pregnancy is continued. In the United Kingdom (UK), around a third of pregnant women (33%) are induced, a rate that has risen from around 20% over the last decade [1]. IOL is a complex medical procedure to initiate cervical preparation, progressive uterine contractions and effacement and dilatation of the cervix. For most, two steps are involved, step one involves administration of an agent to soften the cervix, known as cervical ripening. The second step involves amniotomy (releasing amniotic fluid) and stimulation of uterine contractions via an intravenous infusion.

Typically, in the UK, cervical ripening takes place most often in hospital using pharmacological methods such as prostin pessary, while mechanical methods such as balloon catheter are used for home cervical ripening. Maternity care is midwife-led for women at lower risk of complications, so cervical ripening may be managed by midwives or obstetricians. Over the last 15 years, cervical ripening has increasingly been offered to some women (those seen as having lower-risk indications such as gestational age) as an outpatient procedure, where they have the agent inserted in hospital and, after a short period of monitoring, then return home for a prescribed period of time, usually 24 h, or until labour begins. Some have suggested that outpatient – or ‘home’ – cervical ripening has the potential not only to increase women’s “satisfaction” with IOL but also to reduce pressures on and costs to clinical services [2]. A small study found that women may view home cervical ripening with a mechanical rather than pharmacological method as more similar to normal labour, giving an increased sense of control compared to cervical ripening in hospital [3]. However, research on experiences of home cervical ripening, and IOL more generally, remains limited, meaning there is little evidence informing the provision of care. This study aims to address this gap, focusing on exploring women and their birth partners’ experiences during cervical ripening both at home and in hospital.

## Methods

This research was undertaken as part of the CHOICE Study, a prospective observational cohort study and nested qualitative study (qCHOICE) exploring the safety, cost-effectiveness and acceptability of cervical ripening at home versus in hospital [4]. The qCHOICE study focused on the acceptability of home cervical ripening and experiences of women and their birth partners and of care providers. It comprised a survey of postnatal women’s experiences of IOL and semi-structured interviews with women and birth partners, and interviews and focus groups with NHS staff in five case study sites. The findings reported here relate to the interviews with women and birth partners only; the survey and staff interview findings are reported elsewhere [5, 6]. An audit trail was maintained throughout data collection and analysis to ensure dependability. Researchers kept reflexive notes throughout data collection and analysis, and CY and MH had regular debriefing meetings during the study to discuss findings.

### Data collection

Data collection involved semi-structured interviews with women and their birth partners in CHOICE study sites. Women were invited to take part through the postnatal survey, which was made available to those who had an induction from 37 + 0 weeks gestation in one of the CHOICE study sites during the research period. When completing the postnatal survey, women were given the option to provide details for a research team member to contact them regarding a possible interview. Women’s demographic information was collected as part of the survey and is detailed in Table 1, including their gestation at time of and reason for induction. Consent to contact their birth partners was discussed, and if relevant, they provided their partners’ contact information. Birth partners were then contacted to take part in their own (or if preferred a joint) interview; their demographic information was not collected as part of the recruitment process. The target was to interview 50 women and 25 birth partners, selecting, if possible, for diversity of cervical ripening setting, service and region. Recruitment for the interviews primarily focused on women and birth partners who received IOL care from one of the qCHOICE case study sites; however, some participants from who received care from wider CHOICE study sites were also invited to take part, specifically women who had home cervical ripening and birth partners.

**Table 1:** Demographic and induction information of participants

Age		
Under 25	0%	0/43
25–39	93%	40/43
40–59	7%	3/43
Ethnicity		
White	91%	39/43
Asian/Asian British	5%	2/43
Black/African/Caribbean/Black British	2%	1/43
Mixed/Multiple ethnic groups	2%	1/43
Parity		
Primiparous	63%	27/43
Multiparous	37%	16/43
Gestation at time of induction		
< 39 weeks	14%	6/43
39 weeks	33%	14/43
40 weeks	23%	10/43
41 weeks	16%	7/43
> 41 weeks	14%	6/43
Reason for induction		
Medical reasons (e.g. high blood pressure)	53%	23/43
Length of pregnancy	26%	11/43
Large or small for gestational age	9%	4/43
Spontaneous rupture of membranes	5%	2/43
Reduced fetal movements	5%	2/43
Other	2%	1/43

Interviews aimed to explore participants’ experiences of cervical ripening at home and in hospital, acceptability of home cervical ripening, factors mediating these experiences (for example, information provided, method of cervical ripening, support available) and any unintended consequences. Data collection was undertaken between April 2021 and May 2022 by CY, MH, BC, MF and EP. Two were senior researchers, an anthropologist and a midwife; the others were midwives, including three Master’s students; all had qualitative research training and experience. During this time, some COVID-19 restrictions remained in place and are likely to have affected the experiences of women and birth partners. Interviews were conducted using an online platform and either video- or audio-recorded due to continuing COVID-19 restrictions, as well as participant convenience. Informed consent was obtained verbally and recorded separately before the interviews. Interviews were approximately 60–90 min long, and participants received a £10 gift voucher as a thank-you for taking part.

Interview recordings were transcribed and imported into Nvivo 12 to support data management and analysis. We employed an abductive approach to thematic analysis [7, 8]. This involved an iterative process of analysing data in relation to existing knowledge and ideas about IOL practice in the UK generated from a critical discourse analysis of IOL policy documents and clinical guidelines we undertook during qCHOICE [9]. This allowed for further connections and insights not previously evident and deepened the emergent themes in relation to the wider IOL practice context. Analysis was led by CY and MH, with checks by HC and CM to confirm accuracy. A section of the data was analysed by BC, MF and EP as part of their dissertations under the supervision of CY and CM, and findings from their analysis were incorporated into the larger dataset. Emergent themes were discussed by the research team throughout every stage of analysis.

### Findings

Sixty interviews were conducted with 43 women and 17 birth partners, of which 10 women and three birth partners experienced home cervical ripening. We identified six key themes concerning women and birth partners’ experiences of cervical ripening: ‘Information and choice’; ‘Physical and sensorial environments’; ‘Pain’; ‘Uncertainty’; ‘Care during induction’; ‘Lasting effects’. Findings related to the context IOL care during this study, including information giving, approaches to providing cervicial ripening at home and in hospital and methods of cervical ripening are covered in our previous paper on clinicians’ experiences [6].

### Information and choice

Women and birth partners spoke about information and choice in relation to IOL, with many feeling that they did not have much choice to make. One woman explained that, although she felt she was given an “option”, healthcare professionals led the direction of it:

*I feel that the choices sometimes were given [as] an option*,* but that I was kind of encouraged to go a certain direction. They didn’t force me*,* but I felt that they…(pause) wanted me to…they would say*,* ‘These are the benefits’. They would kind of choose the direction that was more beneficial to maybe the way the ward was working or maybe…what they thought was best practice or what they wanted… If it was reiterated to me*,* ‘This is completely up to you*,* we need to give you some time to make a choice’ and that would have made*,* that would have made a difference. I think my experience would have been a bit better. (Service user 047*,* case study site 4*,* home)*

We found that the number of women offered home cervical ripening was relatively low and tended to be concentrated in some services, where the approach had been established more routinely. Regardless of whether they were in hospital or at home, women often reported a lack of choice when it came to setting:

*[T]he nurse said to us*,* I’m looking at probably five days in hospital. I wasn’t happy about that. I would rather have been at home with my husband. But no*,* I was told that there was no way that I would be able to go home. (Service user 028*,* case study Site 1*,* hospital)*

*I would have preferred to be monitored in hospital*,* but that wasn’t an option. So it was just*,* you know*,* I would be at home with it. (Service user 062*,* case study Site 4*,* home)*

Other participants expressed feeling safer in hospital as a reason for choosing to stay, citing the assurance of being there “to make sure everything’s OK” (Service user 047) and the expectation of constant presence of midwives and obstetricians. An additional concern about going home related to the level of information provided about what to expect with cervical ripening and IOL overall. This woman described her worry as a result:

*Not scared*,* but quite worried*,* that something would happen and I wouldn’t know.* *What I probably didn’t understand*,* would be*,* how painful it would be… What it would feel like during the night when I was at home … no one said to me ‘If you experience this*,* then you should do this. If you feel this level of pain*,* phone us’. (Service user 062*,* case study Site 4*,* home)*

Most birth partners expressed preference for home cervical ripening, so they could stay with and support their partner, but ultimately felt that choices about IOL should be guided by women. Several expressed regret at not being more fully informed:

*I probably should have done more research and I should’ve probably involved myself in the process more to have a greater understanding because if you have the understanding then you don’t have the worry about different aspects. (Birth partner 1*,* non case study site*,* hospital).*

One woman reported not being allowed by the consultant to phone her partner to join the discussion about induction, and her partner described how this affected them:

*I’m angry about it because she wasn’t given an opportunity to make a decision with me or go away and think about it*,* it had to be done in a ten-minute chat with a doctor. We can blame COVID all we want*,* but we’re playing with people’s lives here and big*,* big decisions (Birth partner 4*,* non-case study site*,* hospital)*.

### Physical and sensorial environments

In addition to wanting genuine choice about setting, having their own space was an important mediator to people’s experience during cervical ripening. Women and birth partners reported the lack of privacy in shared induction ‘bays’ contributed to negative experiences, including heightened anxiety and lack of sleep:

*It was awful. Just hearing how long their labours were*,* how sore it was*,* and things that had gone wrong with their baby or their babies are in like the neonatal unit. If they felt like they had lack of choice as well. …It just filled me with fear and dread. And you couldn’t get away from it because you are in a four-bedded room. (Service user 074*,* case study Site 4*,* hospital)*

*[My partner] was trying to sleep*,* and it’s difficult to sleep when people are constantly coming in and out of the room. You know*,* we’ve only got a curtain between you and next door*,* and people are in pain*,* screams. You can hear family on the phone*.* (Birth partner of 097*,* case study Site 5*,* hospital)*

This was mitigated in services where private rooms were available during cervical ripening:

*I think the fact that I had a private room made it much better. I think if I’d been on the ward*,* the experience would have been horrible*,* being in a room with other people*,* because you’re sharing a toilet*,* and it’s just uncomfortable. (Service user 016*,* case study Site 5*,* hospital)*

Those who went home highlighted the benefits in terms of familiarity and comfort of environment:

*It was kind of positioned to me that that was the better option because you get to go home and be in your own house.…It just seemed more relaxed and organised to me. I get to come home and chill and watch TV and have a normal night making tea*,* not take you too far out of your kind of routine… those two combined were really big things for me: going home and passing in my own time. It’s less stress on your partner as well. (Service user 023*,* case study Site 3*,* home)*

This was a widely shared view but nonetheless, some respondents qualified this with other concerns. Those who preferred to stay in hospital emphasised the importance of being in a setting where clinicians were “on hand” to answer questions and provide clinical care:

*I wanted to be in the safety of the midwives*,* doctors*,* nurses and all the care team there. If I’d gone home and anything was to go wrong*,* we don’t live far away from the hospital at all*,* about a ten-minute drive. But it was just really…if I had questions*,* I know that the midwives were there immediately to hand…[I]f I went home*,* I wouldn’t have had that. (Service user 082*,* case study Site 3*,* hospital)*

*[T]he care you’d be getting in hospital*,* they’ve done it a million times*,* so I just feel a bit better when they’re on hand. (Birth partner of 108*,* case study Site 5*,* hospital)*

### Pain

Women reported experiencing pain during cervical ripening, and in some cases this was described as severe. The type and pattern of pain experienced varied depending on the method of cervical ripening being used; some found the balloon more painful during insertion, while others described constant soreness with the pessary:

*It wasn’t that pleasant experience for me at all. I was in a lot of pain having it put in*,* which I did say to them at the time. After the 24 h when they checked they said I actually had a reaction to it. So I was very*,* very sore and swollen. (Service user 030*,* Site 4 – pessary*,* hospital)*

*When I had it done*,* it was as bad as I thought it was going to be. I mean*,* it wasn’t horrendous*,* but it wasn’t as breezy as they kind of made out to be. And it was certainly worse than getting the pessary. (Service user 071*,* case study Site 4 – balloon*,* home)*

Many described being unprepared for the level of pain they felt during the process. More positive experiences were mediated by care focusing on ensuring women were relaxed and comfortable:

*I felt it was ok. I just relaxed*,* the midwives that were dealing with me were just so friendly and they really made you feel comfortable. So*,* I didn’t find it too unpleasant. (Service user 076*,* case study Site 5*,* hospital)*

Some participants perceived home cervical ripening as positively influencing their experience of pain but pointed out a potential drawback concerning the unavailability of pain relief:

*I think at home inductions are really*,* really good idea*,* so then you don’t experience the pain quite as much because you’re in a more familiar environment. I would say it would be a really good*,* but then whether you’d have access to the pain relief that you might require then I don’t know. (Service user 014*,* case study Site 2*,* hospital)*

Yet, expectations of care in hospital were not always routinely met. Several reported they were made to undergo their cervical ripening in hospital without pain relief (which was often connected the type of care they were receiving and is expanded on in the theme ‘Care during induction’), an experience that also negatively impacted birth partners:

*I was in agony*,* it felt like I couldn’t really make any noise because there were three other couples in the room. Feeling spaced out from being tired and from the pain. Then it was about four o’clock in the morning when they eventually gave me some Pethidine. (Service user 028*,* case study Site 1*,* hospital)*

*It was very hard and*,* as I say*,* it was harrowing to sit there and be completely helpless watching this scenario unfold and alerting various people to the fact that she was suffering in real pain. I’ve known [my partner] quite a long time now*,* and I know that she’s tough. To see as much pain as she was in told me that it must have been excruciating*,* and nobody was doing anything about it pain relief-wise. (Birth partner of 028*,* case study Site 1*,* hospital)*

### Uncertainty

Among some who returned home, admission back to hospital and getting into labour ward caused anxiety, especially if they were delayed:

*[G]etting bumped every day because I was a healthy*,* pregnant woman is awfully anxiety inducing. (Service user 049*,* case study Site 4*,* home)*

For others, the benefit of being at home outweighed the potential issue of admission to labour ward:

*If you go*,* then you’re giving up your bed*,* and it might take a while to get back in again*,* but I wanted to go anyway. But then at the same time*,* just because I had a bed doesn’t mean they had space in the labour suite to then proceed. So I could have actually ended up stuck in the hospital for three nights before having the baby*,* which I think would have been worse. (Service user 071*,* case study Site 4*,* home)*

Separation from birth partners was reported during the interviews, often understood as being due to COVID-19 restrictions. The uncertainty of whether birth partners were allowed to be present was raised frequently, especially given their importance in providing support, advocacy and alleviating anxiety during an uncertain time period in an unfamiliar setting:

*I think it’s helpful to have someone else that can be that voice for you and advocate for you and do the things that you’re not able to do*,* because you’re in a very heightened state. Never having given birth before*,* I think I was also really worried about my own health*,* the baby’s health…if you’re really anxious*,* they can be talking to you and you’ve not heard things. So you’ve got someone else there that can kind of hear all that for you. (Service user 056*,* case study Site 2*,* hospital)*

There were a number of uncertainties experienced and encountered along the way, besides concern about their and their babies’ health. One woman outlined her confusion about how the “urgency” of her induction changed throughout the process:

*[T]he sense of apparent urgency had then turned into ‘Well*,* it’s not really that urgent anymore.’ I’m just sitting there*,* and I know that there was a lot of emergencies happening. [P]eople were getting rushed in all the time*,* and I wasn’t a priority*,* which that was completely fine. I don’t want to be a priority*,* but I wondered why I had been a priority. It was sort of confusing*,* but they said that just as long as I was getting monitored and the baby was doing fine*,* then I was fine to wait. (Service user 056*,* case study Site 4*,* hospital)*

Delays were frequently reported, which added additional uncertainty to an already uncertain process:

*[T]he week before women had actually elected to go to other hospitals in [city] because they just could not get a bed for days at the [Site 1] hospital*,* so that was a bit I was worried about*,* about spending days in the hospital. (Service user 001*,* case study Site 1*,* hospital)*

During delays in the IOL process, many participants described entering into a state of limbo, as they waited to for something – continuing cervical ripening or moving to the labour ward – to happen.

*I think a familiar theme from our experience was we would be told something was happening with the expectation that would be imminent. And then it would go on for hours and without any word. (Birth partner to 076*,* case study Site 5*,* hospital)*

Women touched on the uncertainty of what day their cervical ripening would start, as they kept getting “bumped” down the priority list, especially if they were considered ‘low-risk’. This was a source of anxiety and exhaustion when women had been told they needed an induction because their baby was at risk and needed to be born:

*[W]ith them being so busy*,* I got bumped maybe four days in a row for the induction list… I think the week before when I kept getting bumped was absolutely horrendous. It was very difficult for me to deal with*,* mainly just out of worry that I wasn’t being taken seriously or that I was just seen as not important enough to ever make it to that top of that list. (Service user 019*,* case study Site 4*,* home)*

Having personal space with attentive care and their needs met appeared to make women’s experiences better, but these factors did not completely remove uncertainty or lessen this state of limbo, which made one woman feel “trapped”:

*It was just the two of us sitting about sort of just waiting on news*,* and the days were long … there were times where we were in the room for hours on end*,* and I just didn’t know when we would be expecting the next visit and that made me feel a little bit more trapped in that room. (Service user 030*,* case study Site 4*,* hospital)*

### Care during induction

Participants in our study recognised that staff workloads were affecting their capacity to care and contributing to delays during their cervical ripening. They described experiences of taking on the role of a ‘good patient’, who is compliant to the care processes and does not voice anything perceived as difficult:

*I just felt like I had no choice*,* no one to speak to because you’re on a really busy ward*,* I don’t want to come across as a difficult patient. I just feel like I slipped into that patient role so quickly. (Service user 074*,* case study Site 4*,* hospital)*

*I always made sure when I phoned up to be like ‘Oh no*,* it’s fine*,* don’t worry*,* I understand you can’t help these things' and they say ‘Okay*,* if you’re worried just phone us back'*,* ‘No*,* it’s fine'. As soon as you hang up the phone*,* ‘Oh my God*,* I’m so worried!' (Service user 019*,* case study Site 4*,* home)*.

Women’s care during IOL was often defined by whether they were categorised as in established labour, which, in turn, determined whether they were admitted to labour ward or not. Some spoke of being refused certain pain relief during cervical ripening and differences in care between the induction area and labour ward:

*I kept on saying*,* ‘Please*,* can I have some gas and air?’*,* and she was like*,* ‘Not until we go to the labour ward.’ So I was like*,* ‘I need something. This is not right. It’s not right*,* please*,* can you check me?’. ‘Oh*,* you won’t be that far gone yet.’ (Service user 093*,* case study Site 2*,* hospital)*.

*Once I did get to the labour suite with the Oxytocin drip started*,* I did feel really well cared for. I did feel that the staff knew what they’re doing*,* and they couldn’t have been better. They were so empathetic and kind and reassuring. (Service user 074*,* case study Site 4*,* hospital)*

Participants frequently spoke of the supportive and attentive care they received once they got to labour ward. Women did also experience this during cervical ripening; however, it was less common. When they did receive this type of care, it was connected to positive experiences:

*They [midwives] were wonderful. They came in periodically through the night to keep checking his heart rate. I got plenty of sleep*,* but they were there if I needed anything. (Service user 003*,* case study Site 3*,* hospital)*

*Staff were amazing and I think I was on the induction ward for two days and every midwife I came into contact with was just as lovely. (Service user 076*,* case study Site 5*,* hospital)*

*It was all quite relaxed*,* all the staff throughout the whole experience were brilliant*,* they were all really good. They did kind of talk us through everything*,* they told us everything they were doing. So we felt very comfortable and it didn’t get any shocks. (Birth partner to 100*,* case study Site 5*,* hospital)*

In contrast, some women shared experiences of dismissive care during their cervical ripening, feeling they were not being heard and having their pain dismissed:

*[B]ecause I work in the medical field as well*,* I think it was a bit frustrating the fact that it didn’t feel like my voice was particularly being heard. (Service user 014*,* case study Site 2*,* hospital)*

*Because when you’re being told*,* ‘Oh*,* you won’t be active labour yet’*,* yet the pain is excruciating*,* I’m then left with the thought*,* i*f *this is the start of it*,* how am I going to get through this?’. So actually when they did say*,* ‘Oh my God you’re eight centimetres’*,* all I remember thinking was ‘I knew it!’. I knew that something was wrong. (Service user 093*,* case study Site 2*,* hospital)*

Birth partners also experienced this dismissive care, and many of their interviews describe separation from their partners during IOL, despite recognising the impotance of their roles:

*I think birthing partners integral to the the whole experience. We’re there to support the labourer… So we’re not an inconvenience to to the process*,* we should be there as a benefit to the process. So*,* yeah didn’t really appreciate getting sent away [from the hospital] to come back. (Birth partner to 076*,* case study Site 5*,* hospital)*

Other women described an absence of care, sharing experiences of what they felt to be unsafe care during their cervical ripening:

*[W]e feel a bit let down really by the hospital. I was in a lot of pain*,* but they weren’t doing anything about that*,* but it was less about that. It was more about the fact that his heart rate kept dropping and nothing was really done about it… I was getting really upset for about two weeks afterwards. I thought of what could have happened*,* how things could have turned out differently. (Service user 028*,* case study Site 1*,* hospital)*

### Lasting effects

Some participants disclosed difficult or traumatic experiences during their cervical ripening, commonly linked to pain and dismissive or absent care:

*[I]t was a really*,* really traumatic experience for both my partner and I. (Service user 093*,* case study Site 2*,* hospital)*

*I think I was just worried that I was going to be in absolutely horrific pain for hours on end and it was going to be the worst thing ever. That’s still how I remember it*,* but I think probably I’ve blurred my memory a little. (Service user 033*,* case study Site 5*,* hospital)*

Birth partners also reported feeling traumatised after witnessing women in pain and distress and trying to provide support while feeling “helpless”:

*I found it traumatic witnessing it because being completely helpless in a scenario*,* just watching somebody suffer for that length of time*,* trying your best to try and do anything to try and help them out and alleviate that pain in any way you can. (Birth partner of 028*,* case study Site 1*,* hospital)*

Women who had difficult or traumatic experiences reported lasting effects and described how they may shape future care decisions:

*I wouldn’t want to have a C-section*,* but if it came to you need to have an induction*,* I would rather have a section over an induction…[T]here’s no way I would go through that again. (Service user 028*,* case study Site 1*,* hospital)*

These lasting effects of induced labour were often so strong that some participants stated they would need counselling to support future pregnancies or had started therapy as a result of their experience:

*I had a debrief about the actual labour. I’ve actually started some therapy because I’ve not been feeling very good*,* and I do think it’s a fallout from the whole process. (Service user 111*,* case study Site 2*,* hospital)*

## Discussion

This paper describes the experiences of women and birth partners in the UK during cervical ripening at home and in hospital, contributing to the limited literature on the experiences of service users regarding IOL. Although some, in either setting, described a straightforward experience, many were either mixed or negative when it came to the process of cervical ripening, regardless of where it took place. Most women and partners were positive about home cervical ripening as an option and highlighted its potential benefits, yet limited choice about going home and anxiety about returning to the hospital was also reported. Some viewed going home as risky, valuing the constant presence of healthcare professionals, which they expected would be available in a hospital setting. However, our findings suggest that those having home cervical ripening may have over-estimated the level of monitoring or care they would have received in the hospital setting. As described above, this might depend on their location within the hospital, staff workloads and whether the person was deemed to be in established labour or not yet in labour [6, 10, 11].

Many participants experienced uncertainty during cervical ripening, stemming from a number of sources, including the overall process of IOL, setting and partner presence. Our findings concerning process and environment are consistent with previous research on women’s experiences of cervical ripening, particularly feelings of unfamiliarity with the IOL process and hospital setting, lack of privacy, restrictions on birth partners and anxiety [3, 10, 11]. Because induction is primarily offered when professionals consider that continuing pregnancy confers risks to the baby, uncertainty about their babies’ health underpinned many people’s experiences. It was striking, therefore, that a key source of uncertainty were delays in commencing or continuing the IOL process, with consequent anxiety for those at home about admission to the hospital, and for those in hospital about admission to the labour ward. This finding was supported by the wider numbers in our postnatal survey, which self-reported a time period from commencement of CR to admission to labour ward of 3–168 h for those who went home and a maximum of 260 h (11 days) for those who stayed at the hospital [5]. The tension between being categorised as high-risk enough to end pregnancy early but low-risk enough for the process to be delayed, sometimes for days, presented a conflict that profoundly affected some women. We previously identified the ‘low-risk induction’ as a paradoxical concept [6], and participants’ attempted to navigate this, taking on the ‘good patient’ role and complying with professional advice. These findings cannot simply be attributed to current staff shortages as similar findings about candidacy and discordance between women’s needs and operational procedures of clinical settings have been identified in previous studies of IOL experience and of latent phase labour experience [11–13].

Pregnancy is deeply enmeshed in concepts of risk, which are complex and perceived diferently by different parents. Much of the IOL discourse in the UK rests on these concepts, particularly the avoidance of risk of stillbirth [9]. Risk is fundamentally about uncertainty, and its conceptualisation is strongly influenced by social context and norms, and prevalent world views. Our study reveals an incongruity in concepts of and approaches to risk when it comes to IOL. Women are designated as having high enough risk to warrant the necessity – even urgency – of ending their pregnancy but, once the process of cervical ripening has started, are deemed low enough risk to be sent home or experience long periods of waiting on an antenatal ward, often experiencing little support or monitoring. Yet, women know that they have been labelled higher risk and this can become embodied: for example, they begin to feel their body presents a risk to the baby [14]. Many women and birth partners spoke of being in limbo waiting for their care to progress to labour ward and the anxiety this caused, or feeling “let down” after absent or dismissive care. Our study of IOL-related experiences of staff found that they also experienced this dissonance, creating moral distress [6].

Our findings on the limited choice women have regarding cervical ripening in hospital or at home, and IOL overall are consistent with previous research that induction represents a “nondecision”, where lack of balanced information prevents informed choice [11]. They also echo our survey findings, showing consistency with our wider sample where only 66% felt the options were explained in a way they could understand and 57% reported either that they had no choice about having IoL, or no alternative option they could choose [5]. To make a genuine, informed choice, people require sufficient information to have understanding of available alternatives, the opportunity to weigh up options and make a choice that is consistent with their individual values. With respect to location of CR, in our survey, 43% of those who stayed in hospital would have preferred to go home, while 33% of those who went home would have preferred to stay at the hospital [5]. Choice restrictions were evident not just in terms of setting but also when it came to pain relief, with several participants reporting they or their partners were not offered or were even denied adequate pain relief during their cervical ripening. One woman talked about slipping easily into the “patient role”, emphasising how care does not happen *with* people but *to* people.

The ‘good patient’ has long been described in writing on medical institutions; a role connected to a ‘docile body’ that is malleable and compliant and constructed within and by institutions into an expected medical norm [15, 16]. Research on obstetric violence has shown how women may “perform docility” in order to receive adequate care [15]. Although participants in our study emphasised the caring approaches of maternity professionals, they also described performing the ‘good patient’ role either inadvertently or as a means for moving through an uncertain process beset with delays and understaffing, and at times dismissive approaches. Their advancement through this process also depended on whether they were considered to be in ‘real’ labour or not, similar to experiences of women in the latent phase of spontaneous labour [17]. This left women in limbo, occupying a “liminal state between pregnancy and labour”, highlighted by previous research as an important point for conceptualising IOL, understanding parents’ expereicnes and enhancing personalised care [10].

More positive experiences were reported by participants when they perceived healthcare professionals as attentive and present. Some women shared difficult and traumatic experiences of care that was felt to be unsafe and that had lasting effects. Birth partners also experienced trauma, simply from witnessing these events; more research on their experiences of maternity care is needed to inform services of how to best support them.

However, being at home does not necessarily engender more positive experiences of cervical ripening. Our findings show the value of compassionate, attentive and present care from health professionals, presence of birth partners and having privacy, specifically people having their own space, during IOL. While the latter two are arguably more achievable at home, as our findings here and from previous work describe, this setting is not acceptable for, or available to, everyone [5]. Many participants spoke highly of the supportive care they received from midwives and obstetricians, highlighting how relationality remains the core element of optimal experiences of maternity services.

### Strengths and limitations

This is one of few studies on women’s experiences of cervical ripening in different settings (at home and in hospital) and, importantly, one that includes the experience of birth partners, who are still underrepresented in research on maternity care. While our approach to data collection invited all those who had experienced IOL in the study sites, inevitably responses are self-selective and may not be representative of the whole population. Nonetheless, the interview themes were consistent with the findings of our wider survey sample while providing more depth and nuance in understanding their experiences of IOL care in both settings. The findings from women and partners’ experiences were also concordant with those of the professional interviews [6]. Future research should focus on women and birth partners from under-served communities. COVID-19 restrictions during the study period meant all interviews were conducted online, but our experience was that participants found this approach a positive combination of convenience, personal contact and privacy. Most women had given birth during periods when partner presence was not subject to pandemic restrictions, but it is possible that level of restriction, or concern about the possibility of this, was higher during the study. However, the only report received of a partner being prevented from contact related to a requested telephone call. Overall, the number of people who took part was less than intended. Most women experienced cervical ripening in hospital, and the number of women in study sites offered the option of going home during cervical ripening was lower than expected, affecting the main cohort study sample as well as the survey and interview samples. Further work specifically focusing on home cervical ripening experience may be warranted, as well as research with a specific focus on birth partner experiences.

## Conclusion

Participants reported limitations in choice, in relation to IOL and to staying in hospital or returning home for cervical ripening. While most viewed home cervical ripening as a positive option and described benefits such as greater privacy, comfort and partner support, it was clear that being able to choose was key. Some respondents experienced anxiety in relation to home cervical ripening, preferring to be in a setting where they expected they would receive ongoing monitoring and professional care. Busy services, midwifery staff shortages and inability of services to progress the induction process as intended, led to stress and anxiety in relation to delays in admission, with women feeling they needed to perform a ‘good patient’ role to receive care. While staff support was highly valued and helped to support a more positive experience, this was not consistently available, leading in some cases to distressing or traumatic experiences. Our study suggests that a policy of offering home cervical ripening is acceptable to parents, but with the caveat that there should be genuine, informed choice and sufficient staff and resources to care for the numbers of women who undergo IOL.

## Data Availability

The datasets generated and/or analysed during the current study are not publicly available in order to protect the identities and privacy of individuals who took part. The data are, however, available from the corresponding author on reasonable request.

## Abbreviations

IOL: Induction of labour
UK: United Kingdom
